# From Black Holes Entropy to Consciousness: The Dimensions of the Brain Connectome

**DOI:** 10.3390/e25121645

**Published:** 2023-12-11

**Authors:** Denis Le Bihan

**Affiliations:** 1 NeuroSpin, Frédéric Joliot Institute for Life Sciences (Commissariat à l’Energie Atomique, CEA), Centre d’Études de Saclay, Paris-Saclay University, Bâtiment 145, 91191 Gif-sur-Yvette, France; denis.lebihan@cea.fr; 2 Human Brain Research Center, Kyoto University, Kyoto 606-8501, Japan; 3 Department of System Neuroscience, National Institutes for Physiological Sciences, Okazaki 444-8585, Japan

**Keywords:** consciousness, connectome, relativity, spacetime, dimensions, gravity, geodesics, entropy, AdS/CFT, holography, holographic principle, black hole

## Abstract

It has been shown that the theory of relativity can be applied physically to the functioning brain, so that the brain connectome should be considered as a four-dimensional spacetime entity curved by brain activity, just as gravity curves the four-dimensional spacetime of the physical world. Following the most recent developments in modern theoretical physics (black hole entropy, holographic principle, AdS/CFT duality), we conjecture that consciousness can naturally emerge from this four-dimensional brain connectome when a fifth dimension is considered, in the same way that gravity emerges from a ‘flat’ four-dimensional quantum world, without gravitation, present at the boundaries of a five-dimensional spacetime. This vision makes it possible to envisage quantitative signatures of consciousness based on the entropy of the connectome and the curvature of spacetime estimated from data obtained by fMRI in the resting state (nodal activity and functional connectivity) and constrained by the anatomical connectivity derived from diffusion tensor imaging.

## 1. Introduction

In 1998, at the end of a conference organized at the meeting of the Association for the Scientific Study of Consciousness in Tucson, Arizona, Christof Koch of the Allen Institute for Brain Science bet David Chalmers of New York University that a specific signature of consciousness in the brain would be discovered within the next 25 years. In 2023, considerable progress has been made, but a clear understanding of what causes consciousness and how it occurs remains elusive. Most efforts have focused on finding neural correlates of consciousness (NCC), ranging from individual patterns of neural activity (particular types of neurons with special properties) to specific neural networks. A popular model is the global workspace theory (GWS) [1,2,3], which suggests that information from the outside world competes for attention in the cortex, particularly from ‘workspace neurons’ in the prefrontal cortex and thalamus. The information carried by the strongest signal is then sent through the brain via their long-range connections, entering our ‘field of consciousness’. Another network-based theory, integrated information theory (IIT) [4], suggests that consciousness results from the combination of information in a system of specialized modules in the cortex, which are capable of interacting quickly and efficiently. Both models match, in some way, the results obtained in the brain, for example from electroencephalography (EEG), magnetoencephalography (MEG) or neuroimaging, such as functional MRI (fMRI). However, while the GWS emphasizes the critical role of the frontal cortex, the ITT places the NCC in the posterior cortex. Researching the NCC can give us clues as to the *spatial* locations in the brain that are particularly solicited by conscious activity, but the two models do not tell us how consciousness, a subjective experience, emerges from a physical support, the brain. In addition to *spatial* locations in the brain, some researchers have also emphasized the essential role of *time* in the brain, as in the temporo-spatial theory of consciousness (TTC) [5,6].

On the other hand, it has recently been shown [7] how concepts borrowed from Einstein’s theory of relativity [8] can be physically relevant to explain brain function (surprisingly, the application of the concept of relativity to the brain was also briefly considered by Suominen as early as the late 1950s [9]), so that space and time are tightly blended within the brain connectome, which should be considered a ‘flat’ four-dimensional (4D) spacetime entity. This functional spacetime is further curved by brain activity (the activities of neuronal nodes are equivalent to neuronal masses), just as gravity curves the 4D spacetime of the physical world according to Einstein’s theory of general relativity [10]. Pursuing this line and considering the most recent development in modern theoretical physics (anti-de Sitter/conformal field theory or AdS/CFT duality), we conjecture that consciousness emerges naturally from an ‘unconscious’ 4D cerebral cortical connectome when a fifth dimension is considered, just as gravity emerges naturally in a five-dimensional spacetime from a ‘flat’ gravitationless 4D spacetime quantum world present at its boundaries [11].

### 1.1. The Brain Connectome Spacetime

In the universe, the speed of light (c) is a limit and a constant on which Einstein built his theory of special relativity in 1905 [8]. The consequence of this speed limit is that time and space can no longer be considered as separate dimensions, but become intertwined, interchangeable, within a four-dimensional spacetime framework. A second consequence is that mass (m) and energy (E) are equivalent (E = mc^2^). In the brain, the speed of propagation of nerve impulses also has a limit, which we call c*, slower by several orders of magnitude of course, but nevertheless a finite limit. So, what happens if we extrapolate Einstein’s theoretical framework by slowing down its speed limit and applying it to the speed of the brain?

**Figure 1:**
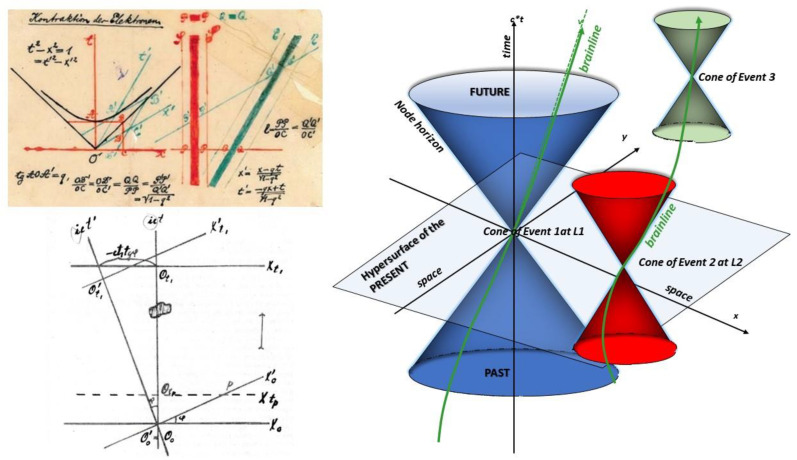
Minkowski’s spacetime translated to the brain connectome. Top left: Figure taken from Minkowski’s 1908 paper [16] showing how space and time (here with only 2 axes, x (horizontal) for space and t (vertical) for time) are blended into a combined spacetime as a consequence of Einstein’s theory of relativity [8]. The 45° oblique lines correspond to the speed of light. Bottom left: Minkowski’s figure has been adapted by Suimonen and reproduced as a figure in his article [9]. Bottom right: Same concept developed for the brain [7], but here with 3 axes (c*t for time and xy for space) and with the oblique lines corresponding to the speed limit of propagation of the cerebral connectome, fixing the boundaries of the events in the cone. An event is a point of ‘localization’ in both space and time. Events are linked in spacetime by brainlines. For a given event, only the brainlines that remain inside the event cone are causally linked (in the past or future), as is the case for events 2 and 3. Events occurring simultaneously (hypersurface of the present), such as events 1 and 2, cannot be linked, as this would imply an infinite speed, greater than the limit. A brainline passing through the same place over time would represent a loop.

This vision was recently presented [7], showing that, like the universe, the brain, or rather the connectome, a set of cerebral areas made up of clusters of neurons (grey matter nodes) and their connections via white matter fibers, sees the dimensions of ‘space’ and ‘time’ mixed up. The light of stars visible at a given moment of the night do not correspond to any reality of simultaneity because they were emitted at very different times, millions or billions of years ago. Time and space merge into a combined spacetime. The connectome’s speed limit imposes the same conclusion for the brain: each given cerebral node ‘sees’ the others only through the nerve impulses it has received from them, i.e., from the ‘past’, if only for a fraction of a second, which implies a different temporal frame of reference for each group of neurons. Similarly, this node will only be seen by the others in the future. This is a radically different and dynamic vision from that given by the usual brain activation maps obtained by neuroimaging, such as fMRI, which are frozen at a given moment like our vision of the starry sky. Instead, we need to consider that influxes propagate along ‘brainlines’, linking in a four-dimensional *spacetime* a series of spatio-temporal *‘events’*, the ‘atoms’ of our brain history, and no longer *spatial* locations within the connectome. Two events can only be linked by their past or their future, because simultaneity would imply an infinite speed of propagation (Figure 1). The result is that, in the brain, the concepts of simultaneity and present become evasive and relative, reflecting the temporal path of innumerable nerve impulses in the spatial tangle of more than 100,000 trillion connections in the cerebral cortex between our senses’ perception of the world and our action in return on our environment. It follows from this concept that any shift in these lines, any delay, due for example to anomalies in propagation speeds, can have major consequences in clinical terms, such as mental illness. This is probably the case in schizophrenia, where diffusion tensor imaging has revealed significant alterations in certain white-matter bundles connecting various brain regions (e.g., fronto-temporal connections) [12]. These anomalies in cerebral spacetime could be at the origin of the auditory hallucinations perceived by the majority of schizophrenics [13]. For these patients, it seems that they hear voices internally *before* the corresponding thoughts are emitted in the prefrontal cortex [7]. There is increasing evidence that the phenotypes of psychiatric disorders are indeed linked to white-matter abnormalities, such as axon diameter affecting conduction velocities, and could therefore be characterized as connective spacetime disorders (see also below) [14,15].

### 1.2. “Gravitation”, Brain Activity, and Spacetime Curvature

After the publication of his paper on special relativity in 1905, Einstein realized that the resulting concept of spacetime (actually introduced by Minkowski in 1908 [16]) was too rigid (‘flat’) and failed to explain certain features of the physical world, notably gravity. This led to his theory of general relativity, which, after several incomplete attempts, was published in full in 1915 [10]: spacetime is in fact curved by the masses present in it, such as stars, which identifies gravitation as a pure effect of geometric curvature. To use a classic metaphor, a ball placed on the edge of a trampoline where we’re standing will roll to our feet because of the curvature of the trampoline’s surface induced by our weight, not because we’re attracting the ball.

Similarly, in the 4D brain connectome, events can also be represented as points in a four-dimensional spacetime where space and time are coupled, with (c*t) appearing as a spatial dimension. Associated with this cerebral spacetime is a four-dimensional metric in quadratic differential form for assigning ‘distances’, ds^2^:

ds^2^:= −c*^2^dt^2^ + dx^2^ + dy^2^ + dz^2^ (1)

where dr^2^ = dx^2^ + dy^2^ + dz^2^ corresponds brain nodes’ spatial coordinates (in the brain space), or more generally, to follow Einstein’s compact tensorial notation (summation convention):

ds^2^ = g_μν_dx^μ^ dx^ν^ (2)

where μ,ν = 0, 1, 2, 3 such that x^μ^={c*t = x^0^, x = x^1^, y = x^2^, z = x^3^} and g_μν_ is a symmetric metric tensor. In a flat spacetime, one has g_μν_ = diag (−1, 1, 1, 1) (the signs may be opposite, depending on the convention used).

In the presence of distributed, dynamic brain activity, the metric tensor g_μν_ in Equation (2) evolves, varying along spatial and temporal coordinates, fully describing the geometric curvature of the four-dimensional connectome spacetime, which becomes curved in the same way that the spacetime of the universe is curved by masses under the effect of gravity. Following Einstein’s approach to establishing his field equations [10], the metric, g_μν_, is derived from the constraint energy content of spacetime as a tensor, as follows:

R^μν^ −1/2 R g^μν^ + Λ g^μν^ = kT^μν^ (3)

where R^μν^ represents the symmetrical Ricci tensor, R the curvature (or Ricci) scalar, and k a normalization constant (the ‘cosmological constant’, Λ, which was not present in the 1915 paper, but was introduced by Einstein in 1917, has been included here for completeness, representing for the brain the interaction between multiple connectomes [7], but we will see later how it might also reflect the degree of consciousness in the brain connectome). T^μν^ is the rank-2 stress-energy (or ‘stress-activity’) tensor describing the distribution and flow of activity in a region of brain spacetime responsible for its local curvature. T^00^ is the local activity (in terms of energy) in the nodes, while the other terms correspond to the rate of activation flux in spacetime around the nodes. Using a pseudo-diffusion model [7], the level of node activity can be related to a pseudo-diffusion coefficient, D*, with

D* = ω/4ρσ(4)

where ρ is the density of nodes, σ is the ‘cross-section’ (probability of diffusing activity reaching a node), and ω is the average “collision” rate (firing) within the network. D* would depend on the balance between local information processing within clusters (high ρσ) and global information transmission via long-distance connections. As for the curvature of connectome spacetime, to some extent T^μν^ would be related to D*, with, at each brain spacetime event, T^00^~ρσ (local node energy density) and T^ii^ ~ω (’pressure’ or the energy transferred per unit area and unit time in all directions by the ‘thermal motion’ representing the flow of pseudo-diffusive activity).

Just as light follows the curvature of spacetime imposed by massive stars, we can consider that the brainlines followed by nerve impulses in cerebral spacetime follow a kind of functional curvature induced by the activity (energy) of all the cerebral nodes that are equivalent to masses, minimizing their trajectories in the spacetime of the combined connectome, just as flights connecting London and New York have a curved trajectory over Greenland, following the curvature of the Earth. Brain activity therefore flows in the brain’s spacetime along ‘geodesics’, i.e., ’straight’ lines in this four-dimensional curved pseudo-Riemannian space. The concept of *path length* (frequently used in network models), defined as the *shortest path* between two nodes, will now have to be considered in this four-dimensional spacetime geometry, with the temporal dimension becoming part of the path.

**Figure 2:**
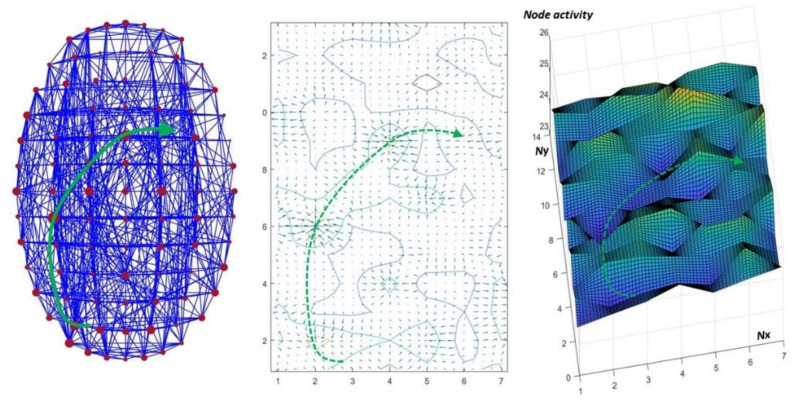
Curved connectome spacetime, mental landscapes, and geodesics. Left: The curvature of a 2D brain spacetime (here, only two dimensions are used for space, one for time) is caused by the activity of nodes represented here by the size of the dots in red. Nodes in the network were randomly connected to all other nodes with a speed limit corrected by the length of the connections. The activity level of each node was set randomly and modulated by the activation flow (probability of hitting a given node). Center: The corresponding activity flow model can be represented by vector maps (fields) and contour lines (potential lines) showing the spatial regions where activity flow converges. Right: These maps can also be represented by three-dimensional graphs where the third dimension reflects the activity level of each brain region (the lowest points corresponding to the most active nodes, and vice versa). These graphs represent the curvature of this three-dimensional “landscape” of cerebral spacetime, with activity flowing geodesically from peaks to valleys (adapted from [7]).

It is worth noting that, just as in the universe the propagation speed of light (as seen by an observer) varies in a spacetime curved by masses [17], the propagation speed of the action potential is expected to vary between geodesics. Indeed, this speed is not uniform within the connectome, but scales linearly with the thickness of the axon myelin sheath and the length of the axons [18,19]. Interestingly, propagation speed is slower for short connections, i.e., between nearby nodes, and faster between distant nodes. This mirrors, in a way, what happens in the physical universe: light travels at full speed away from masses and slows down near masses. Note that the information carried by action potentials is encoded in frequency, not propagation speed. There is a beautiful analogy here with light, which propagates at finite speeds, but with photons of different frequencies (colors). In short, we can consider that trains of action potentials carry ‘colors’. Neurons can fire fewer action potentials in a given time interval (lower frequency, ‘reddish’) when the conduction velocity is low due to the refractory period; in a similar way, light gets redshifted in a gravitational field around masses. As a result, the shortest paths may no longer be associated with the shortest physical distances. Obviously, action potentials follow anatomical axonal pathways, as shown by diffusion tensor MRI (DTI), but the relevant pathways between events must nevertheless be as close as possible to these geodesics, minimizing both space and time: Instead of a direct connection between two nodes, a functional connection may require a more complex pathway of connections involving several nodes (Figure 2), combining the physical geometry of the brain with the dynamics of propagating activity [20]. This is also why DTI data will be extremely useful when applying this framework to the brain, as the fractional anisotropy along the tracks reflects the local amount of myelination, and therefore the local speed of propagation.

The geodesics obey the following equation (using Einstein’s summation convention):

d^2^x^n^/ds^2^ = −Γ^n^_mr_ dx^r^/ds dx^m^/ds (5)

where m,n,r = 1, 2, 3 such that x^n^ = {x = x^1^, y = x^2^, z = x^3^}. Γ^n^_mr_ is the Christoffel symbol (combining partial derivatives, ∂g, of the metric tensor):

Γ^t^_mn_ = ½ g^rt^ [∂_n_g_rm_ + ∂_m_g_rn_ − ∂_r_g_mn_] (6)

Solving Equation (3) means finding the metric tensor g_μν_ (the connectome’s 4D spacetime geometry) for a given connectome activity configuration T^μν^, obtaining the curvature of the brain’s spacetime, and then deriving the related geodesics (brainlines) along which the action potentials flow via Equation (5). This is a tedious task, as it involves second-order partial derivatives of the coefficients of the metric tensor g_μν_ with respect to spacetime coordinates and its inverse, resulting in a set of numerous non-linear equations. In short, to paraphrase JA Wheeler, the famous gravitational physicist: the curvature of connectome spacetime tells neural activity how to flow, while neural activity tells connectome spacetime how to curve. Mental states then appear as configurations, landscapes, of this 4D spacetime whose geometry is permanently distorted, following or preceding the thread of spontaneous or conscious brain activity (Figure 2).

**Figure 3:**
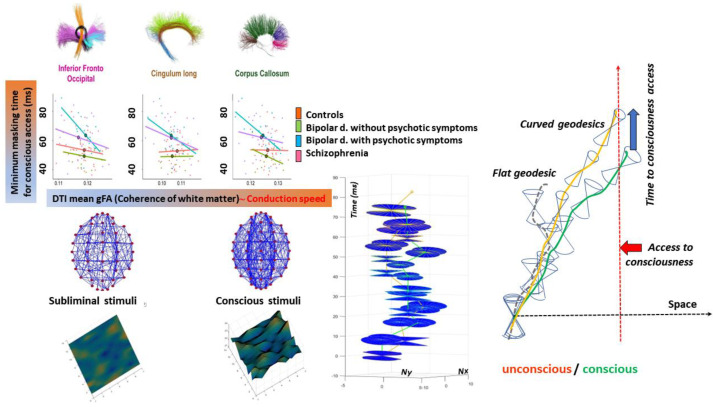
The 4D connectome curvature associated with subliminal and conscious stimuli in normal subjects and psychotic patients. Left: Correlation between the time intervals separating the presentation of stimuli (target digit) and random letters (mask), enabling conscious access to the target (digit recognition), and the level of fractional anisotropy (gFA) obtained from diffusion tensor imaging of 3 selected fiber bundles. When the time interval between the target and masking stimuli is too short, the target is not identified, although it is “perceived” (subliminal stimulus). gFA is a marker of white-matter integrity and is related to the underlying propagation velocity: higher velocities result in shorter masking times. Patients with psychotic features require longer stimulus presentation times before masking becomes conscious (adapted from [15]). Right: Schematic view of the 4D connectome spacetime illustrating how its curvature could allow perceived stimuli to become conscious after a certain time (green curve). Slower speed limits (narrower event cones, yellow curve) translate into longer access times to consciousness. Bottom center: simulation showing how brain lines can reach a target in the brain connectome after a certain delay when speed decreases on certain segments. Interestingly, this simulation also shows that neuronal activity may not follow the same trajectories due to changes in geodesics.

Note here that the term on the left of Equation (5) (the second derivative of a position) has the form of an “acceleration” (*s* is the inverse of the ‘proper time’ in Minkowski spacetime), while the term on the right is a kind of ‘force’ that reflects the underlying metric (the curvature of spacetime). For the universe, this metric is the gravitational field. For the brain connectome, this metric can therefore represent attention or consciousness, which can also be considered as a *field* (not to be confused with the ‘fields’ of neural field theories, which can also be linked to brain geometry [20], or consciousness field theories, which have been used in completely different philosophical, biological, or physical contexts). In short, brain activity curves the spacetime of the cerebral connectome, acting as a kind of force, with consciousness appearing when the curvature reaches a certain threshold. An example can be found in [15], where it is shown that subliminal stimuli are only perceived consciously if they are present for a sufficient interval of time before being masked by other stimuli. This framework may explain how subliminal stimuli ‘entering’ the brain may not reach consciousness. As the level of curvature of the connectome spacetime depends on the speed of propagation, the time required to reach consciousness may vary from one individual to another. In particular, this ‘time-to-consciousness’ threshold can be increased when the speed limit is lowered, for example due to abnormalities in white-matter fiber pathways, as encountered in certain psychiatric disorders (Figure 3).

Asserting that the activity of the brain nodes curves the spacetime of the 4D connectome, imposing geodesics for the propagation of action potentials which, in turn, modulate the activity of the brain nodes, does not explain consciousness per se, but only that conscious activity is linked to the curvature of the spacetime of the 4D connectome, which can be considered a signature of consciousness (we will see later how this feature could be exploited to quantify the level of consciousness). The situation is the same with Einstein’s field equations of the theory of general relativity, which associate the curvature of the universe’s spacetime with gravity from matter but give no clue as to how gravity actually emerges from mass and matter. On the other hand, quantum mechanics has been extraordinarily successful in explaining matter (particles and fields or forces between them) and mass, but has, so far, particularly failed to account for gravity. Similarly, neuroscience has given us an in-depth understanding of the functional organization of the brain, from the molecular level to synapses and neural circuits, but has failed to ‘explain’ what consciousness is and how it physically emerges within the connectome. In the following sections, we will look at how adding an extra dimension to the 4D connectome, in line with the holographic principle, could fill this gap.

### 1.3. From Black Holes Entropy to the Brain Information Content

Another great prediction of Einstein’s theory is the existence of black holes. Black holes are fascinating entities. A black hole is formed when the mass of an object, such as a large star, becomes extremely dense through gravitational collapse. The curvature of spacetime then becomes such that anything entering a black hole can no longer leave it, as it would have to travel faster than light, which itself can no longer escape (hence the connotation ‘black’) once it has crossed a boundary called the black hole’s ‘event horizon’. An interesting question is how much information a black hole can contain.

**Figure 4:**
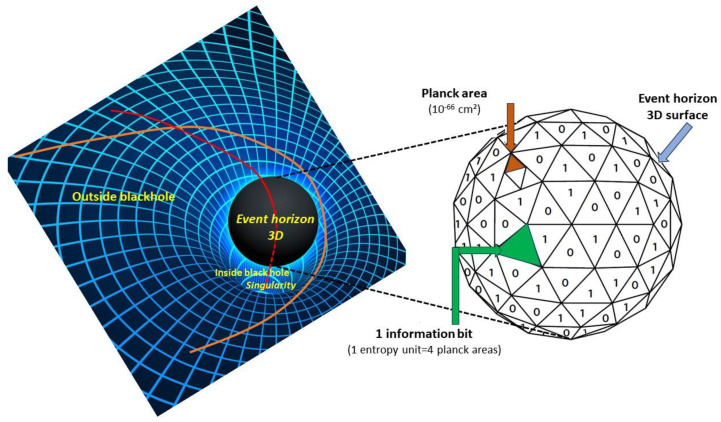
Black hole horizon and black hole entropy. Left: A black hole results from an extreme curvature of spacetime, generally due to the collapse of a massive star under the effect of gravitation, curving light trajectories (orange and red curves). Passing a boundary called the event horizon, the curvature is so strong that there is no way for even light (red curve) to escape, hence the term ‘black hole’, and it falls inexorably towards the singularity at the ‘bottom of time’ (future) of the 4D black hole. The event horizon is in fact a sphere in 4D spacetime. Right: It has been shown that the maximum amount of information (or entropy) a black hole can contain is determined by the 3D surface of its spherical horizon, not by the 4D volume of the black hole, hence a 1-dimension reduction.

Stephen Hawking showed in 1971 [21] that the surface of the black hole’s horizon (Figure 4) can only increase: the more matter or energy enters the black hole, the more its mass increases, and the radius of its horizon with it, knowing that this radius, R, is given by:

R = 2Mg/c^2^ (7)

for a typical Schwarzschild black hole of mass M, where g is Newton’s gravitational constant (for a rapidly rotating black hole, this constant is halved). This gave Jacob Bekenstein the idea that a black hole resembles a thermodynamic system in which entropy only increases according to the second principle of thermodynamics [22,23]. Hawking went further, combining quantum mechanics and special relativity to show that the black hole did indeed have entropy, and that the upper limit of this entropy, S, was directly proportional to the area, A, of its horizon [24,25]:

S = πkc^3^A/(2 hg) (8)

where k is Boltzmann’s constant, and h is Planck’s constant, or simply a quarter of its surface area expressed in Planck units (the Planck unit of surface area is *hg*/2*πc*^3^, which gives 2.6115 10^−70^ m^2^). Given the link between entropy and information quantity established by Shannon [26], this astonishing result tells us that the information content of all objects falling into a black hole is proportional to the surface area of its event horizon and not to the volume of the black hole, with a surprising reduction in dimensions from three to two (although it should be remembered that, in fact, the horizon of a 4D spacetime black hole is a spheroïd, so the reduction in dimensions is from 4 to 3) [27].

Surprisingly, the reality of this dimensional reduction of the world and the universe is echoed in the way the brain is anatomically structured. A large proportion of the brain’s neurons (fourteen to sixteen billion) are concentrated on the surface of the brain, the cortex, a thin, highly wrinkled band two to four millimeters thick in humans, extending over some 2800 cm^2^. The subcortical volume is occupied mainly by the nerve fibers making up the white matter (with the exception, of course, of the neurons present in the basal ganglia in the center of the brain). In the cortex, the average density is around 18,000 neurons/mm^3^ (to which must be added at least as many glial cells). In the fetus, neurons are produced in the center of the future brain, around the neural tube, from where they migrate in a complex process. If, instead of this migration, neurons were to remain and accumulate in the center of the brain, gradually filling it to a final size of around 1300 cm^3^, we would obtain—with the same neuronal density (and therefore taking into account the presence of other cells, such as glial cells) and considering the brain as a sphere—a capacity of almost three hundred billion neurons. This fact is already a clue that the brain shares a common characteristic with a black hole: its information content seems to be distributed over its surface rather than its volume, with a reduction in spatial dimensions from three to two.

In a ‘gedanken experiment’, we will consider what would happen if we could massively compress our brain (which we will give a mass of 1.35 kg and approximate to a 1300 cm^3^ sphere) until it reached the critical density needed to become a black hole. The size of its horizon (radius) would then be 2 × 10^−27^ m, and its surface area 2 × 10^17^ Planck units (given this tiny size, it would evaporate almost instantaneously due to its quantum fluctuations, but we will ignore this tragic fate). According to the Bekenstein–Hawking equation (Equation (8)), the maximum amount of information it can contain is 6.95 × 10^16^ bits, all of which is localized on this surface. On the other hand, assuming that each synapse of a dendrite encodes one bit (active or inactive) of information, with an average density of 8 × 10^8^ synapses/mm^3^, our cortex can indeed handle up to 6.72 × 10^16^ bits, or 8.4 terabytes. Although this may be purely coincidental, given the approximations used for these calculations, this estimate suggests that the human brain, as it stands with its cortex, would in fact be physically quite close to its limit of informational capacity present on its surface: here again, it seems that it is the surface area of the brain that counts when it comes to information, and not its volume. Indeed, if we consider the biological evolution of all animal species, the brain’s surface area has expanded more rapidly than its volume would suggest over the course of evolution [28], apparently following this law of physics. At the extreme end of the spectrum, the brain surface is very wrinkled in humans, and even more so in dolphins and whales. It should be noted, however, that the Bekenstein–Hawking equation was established for the ‘smooth’ four-dimensional spherical horizon of a Schwarzschild black hole, the simplest type. However, the high wrinkling of the human brain cortex results in a surface-to-volume ratio around 30%, greater than that of a simple sphere [28]. The capacity of the cerebral cortex is therefore probably greater, on the order of 10 to 15 terabytes.

### 1.4. The Holography Principle at Play in the Connectome

This recent concept of dimensional reduction, from a ‘volume’ of any number of dimensions to its ‘surface’ limits (with one dimension less), and vice versa, seems to constitute a major breakthrough in physics. This view was taken up by Gerard’t Hooft (Nobel Prize, 1999) in the form of the holographic principle [29,30] and generalized by Leonard Susskind in the context of quantum string theory [31]: “The three-dimensional world of ordinary experience—the universe filled with galaxies, stars, planets, houses, rocks and people—is a hologram, an image of reality encoded on a distant two-dimensional (2D) surface”. Today, holograms are common flat images that give the impression of being three-dimensional, especially when viewed from different angles. A hologram contains all the information of a three-dimensional object in a two-dimensional image. Technically, the image is formed by illuminating the object with coherent light, such as that from a laser, which serves as a reference for the light reflected by all points on the object. By re-illuminating the flat image, we reconstitute the light that had been reflected by the object and see it reappear as if it were right in front of us, revealing all its three-dimensional details, even though it remains, in fact, an illusion.

Conceptually, the holographic principle explains the existence of a precise and general limit to the *information content* of spacetime regions, *the covariant entropy bound*, which is linked to the *geometry* of spacetime, stipulating that all the information required to describe the physical properties of an object (its particles, and their evolution and interactions) is entirely described, encoded, on its *surface* [32]. The three-dimensional vision we can have inside a volume is in fact only the holographic projection of what lies on its boundary surface, and therefore a kind of *illusion*. In other words, it is the observers (or rather, our minds) who bring the universe into being from the information we perceive. After all, as Eddington put it, “Physics is a description of the world as we perceive it; the matter of the world is the matter of the mind (Sir A.S. Eddington, Cambridge, 1939)”. We can immediately see the relevance of this point of view to consciousness: for an ‘external’ observer, all the information we carry would actually be stored on a two-dimensional surface, the cerebral cortex, whereas for the ‘internal’ observer (our mind), an inner world could be dynamically reconstructed from this information with an extra dimension.

In fact, the three spatial dimensions we believe exist in the world enter the cerebral cortex in the form of multiple two-dimensional ‘images’, i.e., with reduced dimensions, in line with the holographic principle. Cortical areas are associated with particular functions (motor skills, vision, hearing, etc.), but they are organized according to a very particular spatial representation of the world and our body, an organization that is repeated at different scales. The world we perceive through our bodies is entirely projected along these areas, even if this projection is distorted—the size of each cerebral area depends on its evolutionary links with the environment, to better perceive it or act upon it (humans have large areas dedicated to the hands and lips)—but it is extremely precise. In other words, *physical space* (the outside world as well as our own body) is encoded in the brain’s architecture along the *surface* of its cortex, as illustrated by Penfield’s *homunculus* [33]. Clearly, the correspondence between *physical space* and cortex architecture is two-dimensional: the third spatial dimension of the world we *perceive* is *not* encoded in the thickness (third spatial dimension) of the cortical layers. The layers of the cortex are used to segregate the features we perceive from the environment, not to encode a third dimension of space. The best example is found in the primary visual cortex, V1, as shown by David Hubel and Torsten Wiesel in cats and monkeys in the 1960s [34]. As well as being organized into six parallel surface layers, the cortex is also divided perpendicularly into columns of alternating ocular dominance of left and right eyes, spaced half a millimeter apart in the human brain. It is a kind of mosaic in which neurons are grouped together to form functional circuits, processing information hierarchically in the visual cortex.

**Figure 5:**
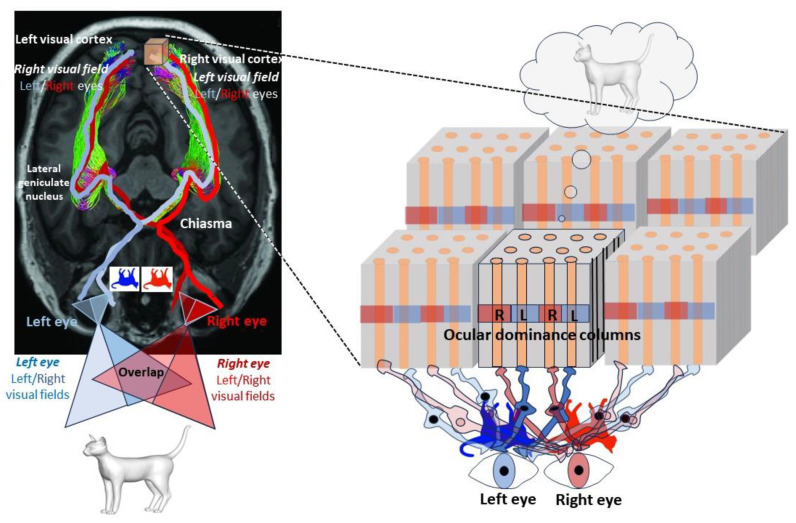
Architecture of the visual cortex. Left: The world we perceive is a simple 2D projection on the retina of both eyes. What we see on the right (right visual field) of both eyes is transmitted to the left visual cortex, and vice versa. To achieve this, the optic nerve fibers that leave the retina cross in the chiasma at the base of the brain, but around 50% of the fibers do not cross in primates. This means that the right visual cortex receives signals from the left visual field of both eyes. Right: Visual fibers from the lateral geniculate nucleus, a relay via the optic radiations, end up in the visual cortex, which takes the form of a mosaic of columns coming alternately from each eye and representing a tiny part of the visual field. Mismatched overlaps between the fields of vision perceived by the two eyes due to parallax leads to the emergence of a sensation of three dimensions, which is therefore a construct of the mind, and hence a kind of illusion.

Just as the motor cortex has its *homunculus*, the visual cortex is *retinotopic*: the first neurons receiving information from the retina (via the optic nerve, the chiasma, and a relay, the lateral geniculate body), located in the fourth layer, are distributed over the *surface* of the visual cortex, each field representing a small part of the *two-dimensional visual space*, as seen by each eye (Figure 5). It is the comparison of the information present in the two columns that gives us the sensation of a ‘third’ dimension, thanks to the parallax effect resulting from the distance separating our eyes: the closer an object is, the more the 2D images projected onto the retina differ. This is possible because the visual field seen by the two eyes partially overlaps, and because the retina of each eye sends fibers to the visual cortexes of both hemispheres via a partial crossing of the fibers (decussation in the optic chiasma) at the base of the brain. While around 50% of fibers do not cross in primates, some animal species (the best example being fish) have complete crossing (in addition, the visual field of the two eyes does not overlap), which prevents them from having a visual perception of the world in three dimensions. The three-dimensional representation of our visual world is therefore a construction of our mind, *adding a third dimension* to the perceived information encoded in *two dimensions* along our visual cortex, a true holographic output, requiring access to consciousness.

Furthermore, given that a phase and an angle are mathematically equivalent, we can say that the emergence of the third visual physical dimension results from the differences in phase between the retinotopic maps coming from each eye, which depend on the varying angles of parallax between the two lines of sight arriving at the left and right retina (convergence of sight), a kind of interference at the heart of the principle of holography.

As far as hearing is concerned, the sounds perceived by the surface of the eardrum in each ear are in some way ‘Fourier transformed’ along the basilar membrane of the cochlea, which is projected topographically onto the primary auditory cortex of each hemisphere, thus producing a 1D tonotopic (frequency) map. The auditory cortex is also organized into orthogonal bands, with this second dimension receiving information from the other ear, in a similar way to the ocular dominance columns of the visual cortex. The 3D spatial perception of sound is again constructed from this 2D organization of the cortex, by comparing the signals perceived by the two ears, not only on the basis of differences in sound intensity, but also on the basis of phase shifts in the propagation of sound waves produced by the presence of the head between the two ears. The representation of a third dimension by phase shifts makes the comparison with holography even more relevant, with the interesting observation that the third spatial dimension is now reconstructed from a temporal dimension. This is also how bats perceive the third spatial dimension from the sounds they emit, which are reflected by obstacles.

The holographic principle can even be applied at a lower, cellular level. It is the central principle of neuronal function: neurons receive signals from other neurons via synapses on their dendritic spines, a kind of outgrowth that increases the *surface* area of the local membrane to a microscopic level. The neuron’s membrane is covered with receptors for specific neurotransmitters and is itself dynamic, since activation leads to local swelling, which locally increases the surface area of the membrane, and vice versa for inhibition. After integration of these multiple signals arriving at the surface of the neuron, the action potentials produced at the emergence cone of the neuron body propagate *along the membrane surface* of the axons, through the white matter fibers of the connectome. In short, all information, whether it enters, transits, or is processed in the brain, occurs at the surface of neurons.

In short, we (our brains) interact with the world solely via the receptors that cover the *surface* of our body (this is also true for internal organs) and the musculoskeletal system that gives shape to this *surface*. All exchanges of information with the environment therefore reach us via the *surface* of our body, including our cognitive relationships with others, since there is, for the moment at least, no direct relationship from brain to brain (nor any direct transformation, in relativistic terms, from one cognitive frame of reference to another, as stated in [35]). This point of view is echoed in the “interface theory” proposed by Donald D. Hoffman [36]. This does not, of course, rule out communication without language, via body posture and above all facial expressions (the motor neurons of the facial muscles occupy the largest space along the motor cortex). But this remains *surface* communication, involving a *reconstruction* by our mind. For example, our mental representation of the other person—of what they might be like inside, of their possible feelings towards us—is only a projection, a construction of our mind (hence the *theory* of mind [37]), and therefore enormously biased by our upbringing and prejudices; we only have access to the surface of this other person, be it their facial expressions, skin contact, posture, or even their words, which we pick up thanks to the vibrations of the tympanic *surfaces* deep in our ears, and vice-versa, in a kind of ‘relativism’ (here in the philosophical sense).

Like the shadows in Plato’s cave, our consciousness is capable of giving us mental images of extremely sophisticated objects or beings, with their emotional status expressed from this surface information. But the process is reversed here, since in this case, reality *is* the surface information, the mental reconstruction not existing outside our own mind, as a reminiscence of the holographic principle of physics that underlies the functioning of the Universe. Remember how it felt the first time you saw yourself in a mirror, or heard your recorded voice, and said to yourself: “Is that *me?*”. In those moments, the duality of our existence jumps out at us, as the multidimensional (and usually flattering) mental image we have of ourselves does not match the authentic, two-dimensional external image that suddenly appears. Nevertheless, it is all that others see or hear of us to judge, appreciate or reject us. In a way, if ‘real’ interactions with the physical environment end up on the surface of the brain—as an outside observer would see it—it is nonetheless true that, from the inside, our own view of the world can be radically different, yet equally acceptable or realistic according to the principle of duality.

Returning to the 4D connectome, the question is then to understand how this inner vision, i.e., consciousness, physically emerges from the ‘information’ present on the 4D surface of the brain, as a kind of hologram of the cortex’s contents. The idea that consciousness (or the mind) is a hologram is not new from a philosophical standpoint—it was proposed by Karl H. Pribram as early as 1969 (holonomic brain theory), but in the context of quantum mechanics [38]. Pribram’s neural holograms are formed by diffraction patterns created by local oscillating electric potentials (waves) in small neural networks, hence, not from the action potentials propagating within the whole connectome, as depicted with the ‘gravitational’, relativistic mind. A common view, however, is that information storage (memory) is non-local, allowing some brain function features to be preserved after some brain areas have been damaged. The hypothesis of quantum consciousness has also been evoked more physically by well-known physicists Roger Penrose [39] and David Bohm [40]. More recently Uziel Awret also attempted to find a physical link between consciousness and the physics of spacetime and information through the ‘strange metal theory’ [41].

### 1.5. The Five Dimensions of the Connectome and the Emergence of Consciousness

The removal (or addition) of a dimension makes it possible to better integrate the different scales of the universe, from the infinitely large to the infinitely small, where gravitation and quantum mechanics apply, respectively, although they remain incompatible today. In 1919, Theodor Kaluza suggested to Einstein that his theory of general relativity could naturally be merged with electromagnetism (Maxwell’s theory) if a five-dimensional spacetime were considered [42]. Quantum mechanics were added to this five-dimensional spacetime by Klein in 1926, a model known as the Kaluza–Klein model [43,44], which is enjoying a striking revival with modern physics. Thorn also observed in 1978 that string theory admits a lower-dimensional description from which gravity emerges holographically [45,46].

This point of view, taken up by physicist Maldacena at a conference in 1997 and in a subsequent paper that became the most cited in theoretical physics with over 20,000 citations [11], is considered the most important breakthrough in theoretical physics of the last 30 years. Based on M string theory including quantum gravity (known as supergravity), Maldacena rigorously demonstrated the correspondence between a five-dimensional anti-de Sitter (AdS) spacetime (solution of Einstein’s field equations [3] with a negative cosmological constant, Λ = −6/L^2^, where L is the anti-de Sitter radius) and a four-dimensional version of quantum field theory (conformal field theory, CFT) excluding gravity. In this AdS/CFT correspondence framework (this is the historical name, it has also been called holographic theory or gauge/gravity theory), the metric of Equation (1) can be written as follows:

ds_5_^²^ = Ω(w)^2^ ds_4_^2^ + dw^2^ (9)

where ds_4_^2^ corresponds to the 4D ‘boundary’, ‘flat’ 4D spacetime (~ds^2^ in Equation (1) which does not include gravity) where a large-Nc gauge (quantum) theory lives, while ds_5_^2^ is now the 5D ‘bulk’, ’curved’ spacetime metric (with an additional dimension, w, which is equivalent to a length) where a gravitational theory lives. For scale invariance reasons the function Ω(w) can be uniquely determined as e^−2w/L^. Posing r = Le^−w/L^ as a coordinate, Equation (9) becomes:

ds_5_^²^ = (r/L)^2^ ds_4_^2^ + L^2^ dr^2^/r^2^ (10)

This 5D AdS spacetime (with its quadratic differential form metric ds_5_^2^) is, thus, formally related to a 4D scale-invariant gauge (CFT) theory (metric ds_4_^2^), such as the Nc = 4 super Yang–Mills theory, 4 being the number of symmetries, when r<<L (near boundaries region). Equation (10), hence, describes a framework where a curved 5D AdS (gravitational) spacetime is embedding a flat 4D (quantum gauge) spacetime, the curvature (∝1/L^2^) occurring with the fifth dimension.

Briefly, Maldacena demonstrated how gravity (and its associated relativistic curvature effect) could emerge naturally in a 5D spacetime from quantum matter, from the gravity-free 4D spacetime at its boundary. These 4D and 5D spacetimes are equivalent and describe the same physics from different perspectives. In a way, the ’volumetric’ content of this 5D spacetime, which includes gravity, is a hologram of the ’surface’ content present at its borders, which is a gravity-free 4D spacetime. Since then, these results have been extended to many areas of physics, notably for a de Sitter spacetime (Willem de Sitter’s solution of the 1917 general relativity equation with positive curvature and a positive cosmological constant) more consistent with our universe. In short, the two theories with different numbers of dimensions are in fact identical, allowing one to be used instead of the other depending on the context (duality). While gravity, whose nature differs radically from that of the other fundamental forces (i.e., electromagnetic and nuclear forces), is not included in the standard model of quantum mechanics (3 + 1 dimensions), it appears naturally when 4 + 1 dimensions are considered. This dimensional duality, the ultimate realization of the holographic principle, not only represents a major advance towards the unification of matter, gravity, and quantum mechanics, by merging string theory and quantum gravity, but could also give us clues as to how a *5D gravity-like conscious mind* might emerge from a *4D quantum-like brain connectome*.

**Figure 6:**
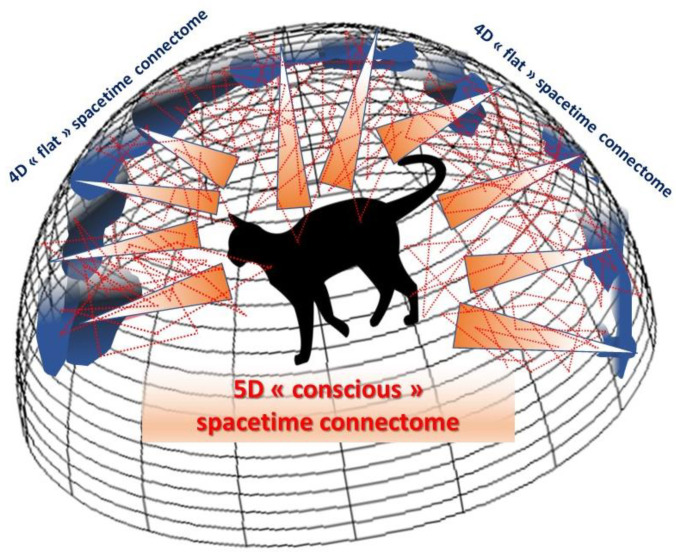
The 5D holographic connectome. Illustration on how consciousness could emerge within a 5D spacetime connectome as a hologram of the information embedded at its boundary, the 4D spacetime of the brain cortex.

Why complicate life with an extra dimension when we already cannot conceive of four, furthermore with the third spatial dimension being itself an illusion, as we have seen? Because this new dimension, physically speaking, makes it possible to explain phenomena that are otherwise inconceivable for a smaller number of dimensions, like ‘2D’ movie actors acting on a flat screen could not explain some of the scenes they play without considering a third dimension (e.g., exiting and entering by two different doors of the same scene). On the basis of these holographic concepts, we can revise the number of dimensions specific to the cerebral connectome, made up of its *material* network of nodes and connections, and their link with the external world we perceive and the internal world we construct, in other words our *mind* and consciousness. We have seen that the third spatial dimension is a neural construct emerging from the visual cortex and resulting from the combined processing of separate 2D information in the ocular dominance columns. If we move to the level of the whole-brain connectome, we immediately see the relevance of this viewpoint in explaining how consciousness could naturally emerge along an extra dimension from information embedded in the 4D cerebral cortex, just as gravity emerges from a 4D quantum universe. This means that the connectome should rather be considered as a 5D spacetime to include consciousness. In other words, a 5D (conscious) curved spacetime can be seen as a holographic image emerging from an ‘unconscious’ 4D flat cortex, a distant hypersurface on which holographic data can be stored and processed in accordance with what neuroscientific theories have explained, in terms of node activity and local neural networks (Figure 6).

This illusory reconstruction by our brains must result from the synchronization of information exchanged between different brain areas via the white matter of the cerebral connectome, as in a standard hologram. On a holographic film, only patterns, blobs and curves that make no sense are visible to the human eyes. To reveal the contents of the object in the form of a three-dimensional image, it is necessary to create a relationship between the different patterns present on the film, obtained by illumination with light whose photons are highly coherent in phase, as laser light allows. These photons, following straight lines from the light source like filaments, will cross the film and be affected in their trajectory (in intensity and phase, i.e., with tiny shifts in time) by the patterns they encounter. By recombining, these photons, which now create interfering waves, will recreate a virtual three-dimensional image of what was recorded on the film. The recreated (but illusory) object appears to be suspended in mid-air, revealing all its details, down to the microscopic level, depending on the angle from which it is viewed.

Similarly, the information processed by the brain takes the form of ’patterns’ in the cortex, divided into multiple functionally specialized nodes. These nodes are all connected, physically and functionally, by the fibers that make up the underlying white matter. The trains of action potentials that propagate in these areas are extremely well defined in terms of *intensity* (frequency) and *phase* (temporal relationship), along the brainlines of the relativistic connectome. We can therefore consider them as the ‘light lines’ of holography which, illuminating the activity of the various nodes of the cerebral cortex, reveal the coherence of extremely precise activities in the spacetime of the connectome, to the millisecond, while obeying the relativistic principle, creating in turn ’images’: what we believe we see, hear, touch, including our feelings, emotions, ideas, thoughts—in short, our consciousness—appears as a dimensional emergence of the connectome. A consequence of this view is that there can be no ‘zone or center of consciousness’ in the brain, since consciousness results from the generalized activity of distributed cortical networks involving both gray and white matter, and not from a single network node in a finite time window (this does imply, however, that there are no neural ‘switches’ to consciousness, see below).

### 1.6. Quantifying and Restoring Consciousness

**Figure 7:**
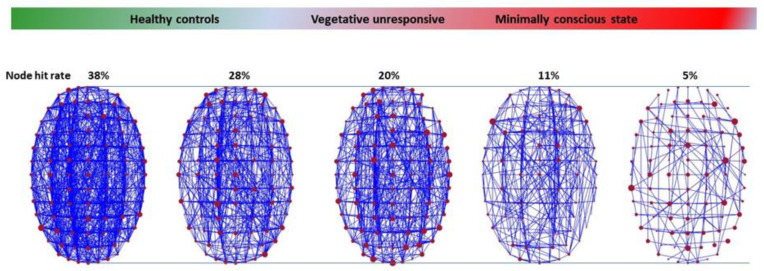
Level of consciousness and spacetime curvature of the connectome. When curvature is reduced (r/L small in Equation (10)), the cerebral connectome reduces to a flat 4D spacetime with sparse, isolated node activity in the cortex and long-distance, fast connections (state of minimal consciousness). As curvature (1/L^2^) increases consciousness emerges within the 5D connectome spacetime (more short-distance connections), with a high level of connectivity (high node hit rate).

Indeed, echoing the AdS/CFT duality, while the normal conscious and awake state manifests itself as a dense network of short-range (low speed) functional connections associated with a strong curvature of a 5D connectome spacetime, this network is reduced to an almost ‘flat’ 4D connectome spacetime during anesthesia (which is ‘ironing’ our spacetime landscape) or in patients in a vegetative state (Figure 7). Connections exist, unless the brain is destroyed, but their functional density is low mainly along fast, long-range connections between distant nodes [47]. This view shows that patients in a vegetative state can still express sparse cortical activity in specific regions triggered by environmental stimuli, while lacking a critical level of connectivity to enable them to express consciousness, at least to enable them to interact with their environment, as in the famous case reported by Owen et al. [48].

It would therefore seem interesting to quantify consciousness as a function of the amount of functional connectivity (or curvature) present in the global connectome. Physically quantifying consciousness would in some way provide consciousness with a status more prone to investigation than that of a ‘subjective experience’. Some attempts have been proposed, notably through IIT theory, which quantifies integrated information by a non-negative parameter, Φ, reflecting the complexity of the underlying interconnected structure (intrinsic irreducibility) [4]. The higher Φ, the higher the level of consciousness.

However, the framework of relativistic pseudo-diffusion naturally allows us to go further in the AdS/CFT duality. In Equation (3), the scalar field, Λ, representing the cosmological constant, can be replaced by a cosmological scalar field, φ, which characterizes a background interaction with the particles causing their scattering [49]. In the framework of AdS/CFT duality, Λ is negative and linked to the AdS radius, L, as Λ = −6/L^2^. By formally identifying Λ and φ, we can conceptually consider that the curvature of the 5D connectome could have a link with the pseudo-diffusion coefficient, D*, introduced in Equation (4). D* would therefore appear to be a natural candidate for quantifying connectome curvature, and hence consciousness. On the other hand, there is a relationship between diffusion and entropy, a key concept in information theory, and therefore the level of information exchange within the connectome. In complex networks, entropy has been used to characterize the properties of network topologies, in particular the shortest (geodesic) paths between nodes, making it possible to measure the propagation of information-carrying signals in the network [50], thus merging structure and function. Interestingly, diffusion processes have been used to quantify the interaction dynamics that take place at the top of complex networks [51], including neural networks, in relation to the underlying system [52]. Indeed, diffusion processes can be associated with an entropy rate: a high entropy rate can be linked to efficient diffusion (ease of propagation between nodes) within the network [53]. To begin with, a very simple relationship between excess entropy (relative to a default baseline) and the connectome’s pseudo-diffusion coefficient can be given by following Rosenberg’s classic expression [54]:

S_ex_ = a ln(D*) + b (11)

where a and b are empirical fitting parameters. More accurately, one may calculate entropy from a diffusion equation. Starting from Shannon entropy:

S(p(x)) = −∫p(x)log(p(x))dx(12)

and using the diffusion equation:

p
(
x
) =
1/
√(
4
πD*t) e^−x²/(4D*t)^ (13)

one arrives at (assuming the boundaries (brain size) are large compared to the pseudo-diffusion distances):

S(t) ≈ 1/√π(πlog(4πD*t) + π) ≈√π [1 + log(4πD*t)](14)

The entropy, S(t), thus, grows with D*t.

**Figure 8:**
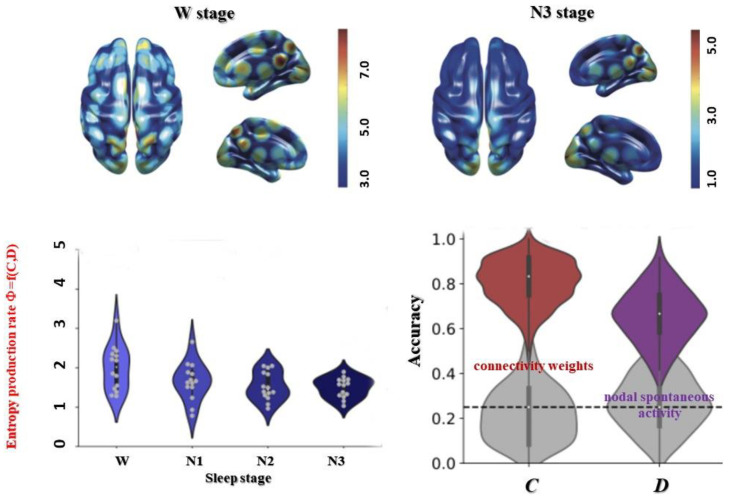
Entropy production as a measure of level of consciousness. Bottom: The entropy production rate obtained from fMRI and DTI data can be used as a marker of the level of consciousness, as shown here for the awake state and different stages of sleep (N1 to N3). This rate of entropy production is calculated from two quantities, C and D, representing connectivity weights and spontaneous nodal activity, respectively, with C contributing more to the prediction of sleep stages from entropy within the connectome. C and D could be formerly related to the stress-energy tensor in Equation (3). Top: The rate of entropy production varies between brain regions, with a higher level in occipital regions during sleep phases (adapted with permission from 55).

Recently, Gilson et al. have introduced a similar approach, although not in a relativistic framework, quantifying an entropy production rate, Φ, within the connectome using a mathematical diffusion framework (multivariate Ornstein–Uhlenbeck stationary diffusion process which is both Gaussian and Markovian) [55]:

Φ = −tr (**D**^−1^
**B Q**) (15)

where **D** is the input covariant matrix (nodal spontaneous activity), **B** a ‘friction’ matrix (propagation of nodal activity, whose off-diagonal elements, **C**, reflect effective connectivity weight between nodes) and **Q** is the irreversibility derived from the zero time-lag covariant matrix **S** as **Q** = **BS** − **D**. An interesting feature of this approach is that **D** and **B**, and, hence, Φ, can be practically estimated from resting-state functional MRI time series, under the topological constraint on **B** of anatomical connections obtained with DTI. The entropy production rate, Φ, is, thus, a scalar measure of irreversibility within the whole connectome network dynamic information process which appears to be correlated to the varying consciousness level occurring during transition from wakefulness to deep sleep, with **C** contributing a little more than **D** (Figure 8).

A limit of the approach proposed by Gilson et al. [55] is that connection speed variations were ignored, as a unique time constant, τ, was used to model effective connectivity and quantify entropy production rate, Φ. Thus, this approach and the framework proposed here might complement each other nicely, taking into account the diffusion process within a 4D relativistic framework [56] and variable propagation speeds within the connectome. This would also bring experimental validation to the present framework, which is still in an early theoretical stage. One may envisage, for instance, that **D** and **C** could be formally integrated into the stress-energy tensor **T^μν^** defined with Equation (3), with T^00^ linked to nodal activity (energy density, **D**) and T^ij^ the ‘energy’ being transferred (effective connectivity, **C**). The consciousness level could then be quantified using DTI-constrained fMRI data (DTI providing the structural connectivity and propagation speed along tracks) by the resulting curvature (Ricci) scalar, R, of Equation (3), or by the quantity (1/L^2^) in the 5D AdS framework (Equation (10)), with high curvature equivalent to a high level of consciousness. In fact, this goes further, as, according to the proposed framework, consciousness, in a similar way to gravity, is nothing less than a spacetime geometric feature, curvature, which does not prevent it, as for gravity, to be perceived as ‘real’ and to manifest itself in a multitude of ways depending on the context, whatever name we give it, such as ‘subjective experience’.

While Φ reflects a global phenomenon, it shows a differentiated magnitude across brain regions, allowing maps to be generated [55]. The occipital regions (cuneus, calcarine, lingual), as well as the posterior hubs (precuneus, postcingulate) remain at fairly high levels of irreversibility despite the decreased level of consciousness associated with sleep. Remarkably, the thalamus retains a high level of irreversibility, suggesting that whole-brain connectivity and associated synchronization may be controlled by ‘switches’ (not to be confused with the NCC), midbrain structures such as the thalamus that control the curvature of a flat 4D connectome spacetime to give rise to 5D consciousness. Such switches could probably also be found in the upper brain stem, which plays a central role in Mark Solms’ ‘hard problem’ of consciousness in the context of the free energy principle, another thermodynamic approach to consciousness [57,58]. So, would it be possible to restore these connections, to bend cerebral spacetime again? This has been demonstrated in an anesthetized rat model [59]. Stimulation of a specific region of the midbrain (ventromedian nucleus of the thalamus), which has numerous anatomical connections and loops with the cerebral cortex, awakened these animals despite being under anesthesia. Similar results have been reproduced in non-human primates, with a clear demonstration of access to consciousness [60]. Clearly, these proofs of concept open up extraordinary prospects for mankind, even if the technical and ethical hurdles remain formidable challenges for the time being.

The fact that the brainstem is probably another key location for such neural switches leads to the hypothesis that the paradoxical (REM) sleep stage associated with a high level of cortical activity and the occurence of dreams could be a solution of Equations (3) and (10), hence, a kind of “conscious” physical state (in terms of high curvature in the 5D connectome) emerging from the 4D cortex connectome, but where interactions with the environment have been mostly or partially deactivated, as also suggested from EEG recordings in napping narcoleptic or healty subjects [61]. Spontaneous events would occur in the flat 4D connectome from information stored internally in cortical areas, instead of external stimuli from the environment, their resulting connections shaping the curved 5D connectome and giving rise to the pseudo-randomness in space and time perceived in dreams, as consciousness is spacetime curvature according to this framework.

## 2. Conclusions

Consciousness thus appears as a ’private’ five-dimensional hologram emerging from the ‘public’ four-dimensional spacetime (accessible to neuroimaging, for example, and responsible for our behavior) of the connectome, with consciousness emerging along the fifth dimension, just like gravity in the universe, from the activity present throughout our cerebral cortex, which is constantly fluctuating, like waves ripping across the surface of the ocean. This point of view remarkably echoes Suominen’s vision, which emphasized over 70 years ago that the consciousness we experience as ‘a single personal self’ is an inner phenomenon (mind-mind), which cannot be dissociated from behavior, an outer phenomenon (mind-body) [9]. In other words, the conflict between ‘consciousness and matter’ could be resolved by considering that the spacetime of our cerebral connectome has not four but five dimensions, the fifth dimension allowing the natural, *immaterial* emergence of consciousness as a dual form of the 4D spacetime embedded in our *material* cerebral cortex.

## Data Availability

No new data were created or analyzed in this study. Data sharing is not applicable to this article.
